# Different Types of Particle Effects in Creep Tests of CoCrFeNiMn High-Entropy Alloy

**DOI:** 10.3390/ma15207363

**Published:** 2022-10-20

**Authors:** Ferdinand Dobeš, Hynek Hadraba, Zdeněk Chlup, Jiří Matějíček

**Affiliations:** 1 Institute of Physics of Materials, Academy of Sciences of the Czech Republic, Žižkova 22, CZ-61662 Brno, Czech Republic; 2 Institute of Plasma Physics, Academy of Sciences of the Czech Republic, Za Slovankou 1782/3, CZ-18200 Praha, Czech Republic

**Keywords:** creep, high-entropy alloy, dispersion-strengthened alloy, spark plasma sintering

## Abstract

Compressive creep tests were performed on a CoCrFeNiMn equiatomic alloy with the dispersion of (i) aluminum nitride or (ii) boron nitride at temperatures of 973 K and 1073 K. The results are compared with previously published creep rates of the unreinforced matrix alloy and the alloy when strengthened by yttrium + titanium oxides. The comparison reveals that the creep rate is essentially unchanged by the presence of aluminum nitride particles, whereas it is reduced by the presence of oxide particles. Boron nitride particles do not influence the creep rate at low stresses but reduce it substantially at high stresses.

## 1. Introduction

High-entropy alloys (HEAs) represent a class of prospective materials with many interesting properties [1,2,3,4,5,6,7,8,9]. As far as their mechanical properties are considered, two phenomena essentially affecting the movement of dislocations, namely, sluggish diffusion and lattice distortion, contribute to the potential application of the alloys at elevated temperatures [10,11,12,13,14]. Equiatomic CoCrFeNiMn, perhaps the most widely studied multi-principal-element alloy, demonstrates a single-phase face-centered cubic solid solution at a relatively wide range of temperatures [15]. Its high-temperature mechanical properties were studied by the authors of [16,17,18,19,20]; creep investigations [21,22,23,24] have been critically summarized and reviewed by Zhang, George, and Gibeling [25]. HEAs with such a lattice structure are ductile but are not strong enough for most purposes. Their strengthening can be achieved by particles being dispersed within the microstructure, which impedes the movement of dislocations. At lower temperatures, dislocations can overcome the particles, either by cutting (which is valid for coherent particles) or by the Orowan mechanism [26]. At high temperatures, the movement of dislocations is facilitated by the possibility of climbing. The increase in creep resistance can then be phenomenologically described by introducing a threshold stress into the dependence of the creep rate on the applied stress [27]. Current theories of the generation of threshold stress are based on two phenomena: (i) an increase in the length of the dislocation line after climbing around the particle [28], and (ii) the detachment of the dislocation from the matrix/particle interface after climbing around the particle [29,30]. In the case of the studied CoCrFeNiMn alloy, the influence of TiC [31,32] and TiN [33] particles on mechanical properties has been studied so far, but only at room temperature. In the following study, the results of creep tests in CoCrFeNiMn alloy strengthened by particles of boron nitride or aluminum nitride are presented and compared with previously published results obtained within the investigation of the single-phase alloy and the alloy when strengthened by the dispersion of oxide particles [24,34].

## 2. Materials and Methods

The AlN or BN nitride-strengthened CoCrFeNiMn alloys were prepared by the mechanical alloying of atomic powders and subsequent spark plasma sintering (SPS). The mechanical alloying was conducted using a planetary ball mill, Pulverisette P6 (Fritsch, Idar-Oberstein, Germany), using a hardened steel milling bowl and steel balls of 1 in in diameter (bearing steel 100Cr6, EN 1.3505). The ball-to-powder weight ratio was kept at 15:1 and the rotational speed of the mill at 350 rpm. First, 100-gram batches of powders composed of equiatomic portions of Co, Cr, Fe, Mn, and Ni (Sigma Aldrich, Saint Louis, MO, USA) and 0.5 g of AlN (hereafter, HEA-AlN) or BN (hereafter, HEA-BN) nitrides (Sigma Aldrich, USA) were milled for 24 h. The composition of the milled batches is provided in Table 1. Milled powders were compacted (SPSed) using the spark plasma sintering technique on a TT SPS 10-4 (Thermal Technology LLC, Minden, NV, USA) at a temperature of 1423 K and a pressure of 50 MPa, with a dwell of 5 min and a heating/cooling rate of 100 K/min. A scanning electron microscope LYRA 3 XMU FEG/SEM (Tescan, Brno, Czech Republic) equipped with an EDS X-Max80 analyzer and an EBSD Symmetry camera (Oxford Instruments, Abingdon-on-Thames, UK) was used for microstructural analyses. The density of the final compacts was determined by double-weighting using the Archimedes method in deionized water and air, following the EN 623-2 standard. The bulk density of the compacts determined by the Archimedes method is provided in Table 1 along with the relative value of the theoretical bulk density, calculated from the nominal chemical composition of the alloys.

Creep tests were performed with uniaxial compression on spark-machined samples with a gauge length of 12 mm and a diameter of 5 mm. Tests were performed on a dead-weight creep machine under a constant load in a protective atmosphere of dry purified argon. The test temperature was kept constant within ±1 K for each individual test. Changes in the specimen length were measured using a linear variable displacement transducer. The samples were subjected to stepwise loading, where the load changed to a new value after the steady-state creep rate had been established for a given load. The terminal values of the true stress and true strain rates were evaluated for each respective step. During one test at a constant temperature, up to 10 changes of the applied stress were performed. The tests were conducted until the strain reached a value of ε = 0.15. The suitability of the stepwise procedure used was verified by a comparison of the obtained creep rates at a given stress level with those resulting from conventional compressive tests under constant stress. During the test, the sample is placed in massive grips (Polanyi grips). Fixing the dislocation microstructure by rapid cooling under load is a complicated process.

## 3. Results

**Figure 1:**
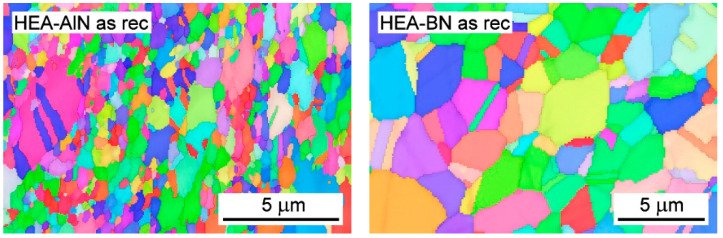
Microstructure of HEA-AlN (**left**) and HEA-BN (**right**) in the as-sintered state.

The results of the energy dispersive spectroscopy (EDS) chemical analysis of the two SPSed compacts are summarized in Table 1. The chemical composition of the sintered compacts corresponds well to the composition of the initial powder blends. A small amount of Si of about 0.2 wt %, originating from the milling balls, was observed. The SEM/EBSD photographs of the HEA-AlN and HEA-BN microstructures in the as-received state are given in Figure 1. The grain size of the HEA alloys varied, depending on the nitride used. The microstructure of the HEA-AlN alloy was significantly finer in grain than that of the HEA-BN alloy. The mean grain size of HEA-AlN was ~0.43 μm, while the grain size of HEA-BN was ~1.53 μm. This was probably due to the lesser effect of BN dispersion on the inhibiting of grain growth during sintering.

**Figure 2:**
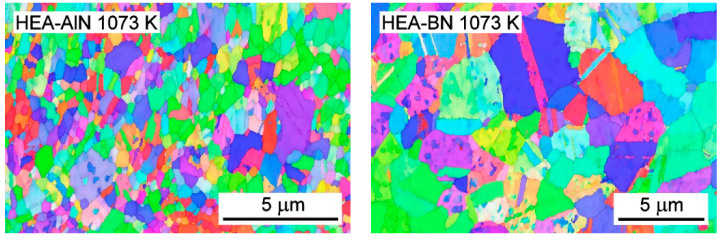
Microstructure of HEA-AlN (**left**) and HEA-BN (**right**) after a creep test at 1073 K.

The SEM/EBSD photographs of HEA-AlN and HEA-BN microstructures after the creep test at 1073 K are given in Figure 2. No major change in the grain structure of the alloys after the creep test compared to the as-received state was observed. The mean grain size of HEA-AlN was ~0.31 μm, while the grain size of HEA-BN was ~1.44 μm. The small variation in grain size after the creep test for both alloys (compared to the SPSed state) corresponds qualitatively to changes in the observed occurrence of twins.

**Figure 3:**
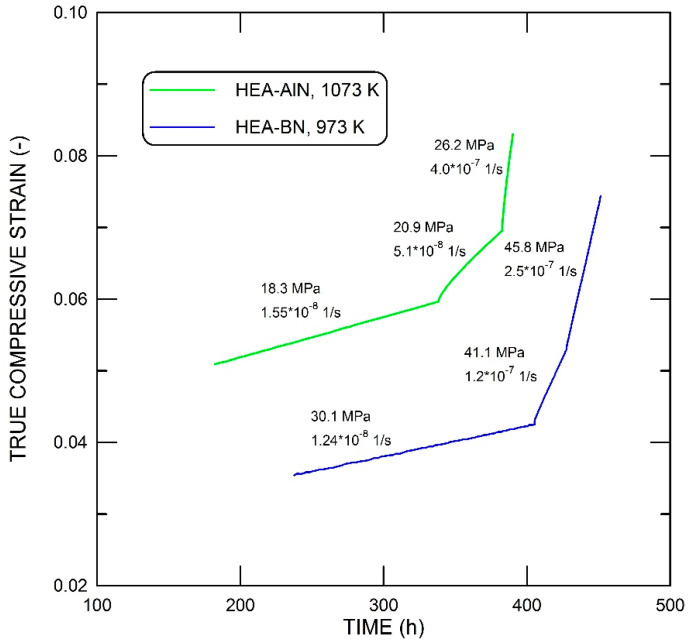
Examples of creep curves at 1073 K (HEA-AlN) and 973 K (HEA-BN). True compressive stress and true compressive strain rates are given for each segment.

**Figure 4:**
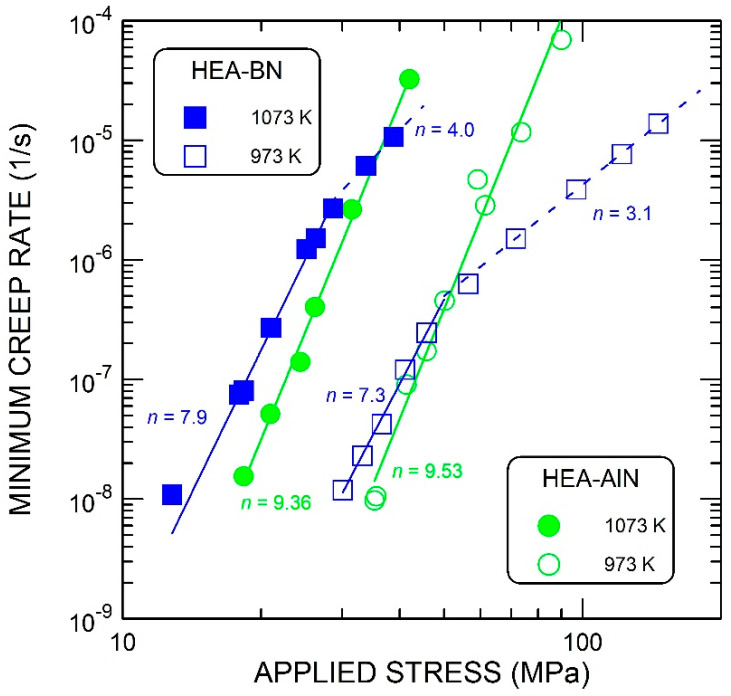
Dependence of the creep rate of the studied alloys on applied stress.

Examples of the time dependence of true compressive strain are given in Figure 3. The transition periods after load changes were quite short and the linear parts of the creep curves were well-defined. The dependence of the creep rate $\dot{\varepsilon }$ on the applied stress σ at two temperatures is shown in Figure 4 on a double-logarithmic scale. For the alloy strengthened by AlN particles, the data can be analyzed using the following relationship:

(1) $$\dot{\varepsilon }=A\sigma^{n}\exp\left(-\frac{Q}{RT}\right)$$

where *A* is a material constant, *n* is the stress exponent, *Q* is the (apparent) activation energy for creep, *R* is the gas constant and *T* is the absolute temperature. The values of *n* and *Q* were determined from Equation (1) by multiple linear regression, assuming that the stress exponent *n* is independent of the temperature: *n* = 9.5, *Q* = 530 kJ/mole.

In the case of the alloy strengthened by BN, it can be seen that the whole stress and creep-rate field investigated can be divided into two regions. At lower creep rates, the dependence on stress and temperature can be evaluated by the same procedure as for the previous alloy with AlN. In this region, the stress exponent is equal to approximately 7.8, and the activation energy is equal to 524 kJ/mole. At higher creep rates, the stress exponent is distinctly lower and is equal to 3.1 at 973 K and to about 4 at 1073 K. The activation energy assessed from the limited available data ranges from 300 to 350 kJ/mole (280 kJ/mole at 20 MPa to 350 kJ/mole at 50 MPa). This is close to the activation enthalpy for the lattice diffusion of individual elements in the CoCrFeNiMn alloy, which range from 288.4 kJ/mole (Mn) to 317.5 kJ/mole (Ni) [35].

## 4. Discussion

**Figure 5:**
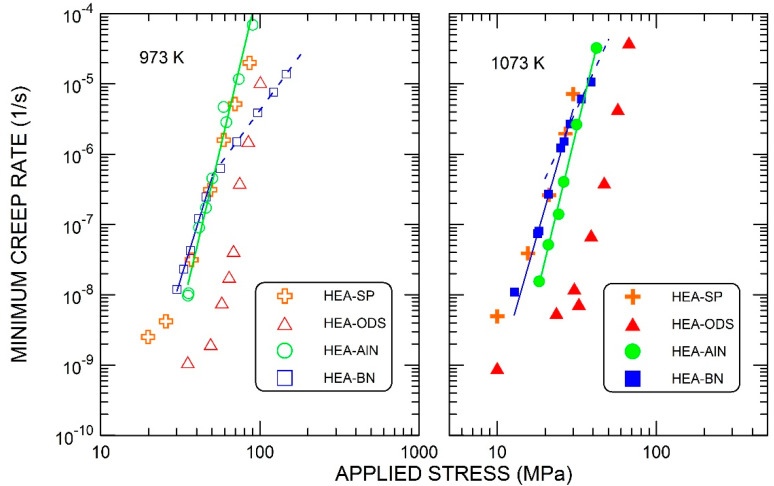
Comparison of the creep behavior of the present alloys, with the single-phase alloy and the alloy strengthened by the dispersion of oxide particles.

The current results can be effectively compared with our previous results [24], obtained for the single-phase CoCrFeNiMn alloy, and for the same HEA strengthened by the dispersion of oxides. The comparison is depicted in Figure 5 for individual temperatures. In the following discussion, the area with very slow creep rates (less than 10^−8^ s^−1^), where the diffusional creep may intervene, will be set aside.

It can be seen that the following categorization of alloys, according to the influence of particles on creep rate, can be proposed:

(i) The creep rate is essentially unchanged by the presence of particles. This applies to the alloy with AlN particles.
(ii) The creep rate is reduced by the presence of particles. This is the case with the ODS alloy.
(iii) The creep rate does not change at low stresses, although it is significantly reduced at higher stresses in the alloy strengthened with BN.

The reasons for the observed distribution into the first and second groups are evidently caused by particle sizes and distances. The particles of the second phase that are embedded in a matrix obstruct the dislocation motion. The stress controlling the dislocation velocity is reduced to an effective value obtained by subtracting the stress necessary for overcoming the particles from the applied stress. Accordingly, the observed high values of the stress exponent (and the activation energy) can be explained by introducing the respective threshold stress $\sigma_{0}$ into Equation (1):

(2) $$\dot{\varepsilon }=A^{\prime }{\left(\sigma -\sigma_{0}\right)}^{n^{\prime }}\exp\left(-\frac{Q^{\prime }}{RT}\right).$$

**Figure 6:**
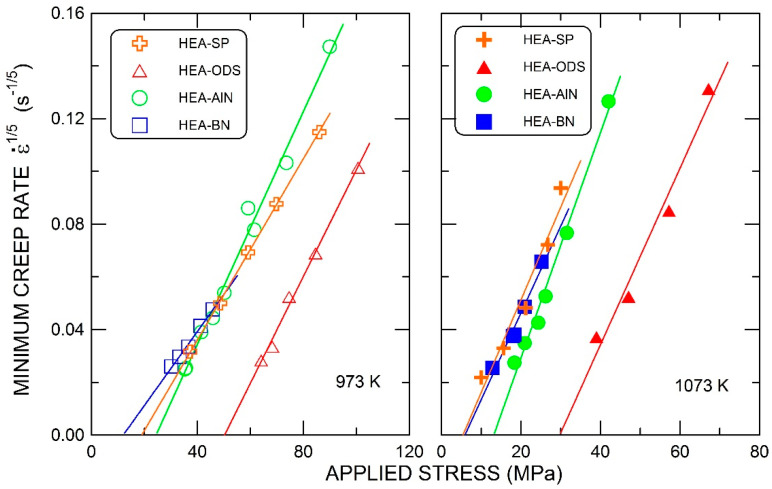
The linear extrapolation method for estimating the threshold stresses using value *n*’ = 5.

The value of the threshold stress can be found by plotting ${\left(\dot{\varepsilon }\right)}^{1/n^{\prime }}$ against the applied stress on the linear axes and extrapolating linearly to a zero-creep rate. The power *n*′, equal to 5, corresponding to the climb-controlled dislocation motion, was used in this determination shown in Figure 6. The estimated value of the threshold stress in the ODS alloy is approximately twice as large as that in the alloy strengthened by aluminum nitrides.

The ineffectiveness of the particles is also evident in the alloy with boron nitrides at low stresses. At high stresses, however, the situation is different. As can be seen from the microscopic observations, a basic difference between the alloy with aluminum nitrides and the alloy with boron nitrides lies in the grain size. The mechanisms that are used to explain the Hall–Petch relationship between grain size and yield strength cannot be used in our case because the observed effect on the creep resistance is completely opposite. On the other hand, the observed effect of grain size corresponds to the usual phenomenological description of the high-temperature creep rate in the steady state (e.g., as used by the authors of [36]):

(3) $$\dot{\varepsilon }=\frac{A^{\prime \prime }DGb}{kT}{\left(\frac{b}{d}\right)}^{p}{\left(\frac{\sigma }{G}\right)}^{n}$$

where *D* is the appropriate diffusion coefficient, *G* is the shear modulus, *b* is the length of the Burgers vector, *k* is the Boltzmann constant, *d* is the grain size, *p* is the exponent of the inverse grain size, and $A^{\prime \prime }$ is a dimensionless constant. For the exponent, *p*, Langdon [36] deduced a value of *p* = 0.9 for large grains and *p* = 2 for small grains in a unified model of grain boundary-sliding in creep and superplasticity and demonstrated that the value *p* = 1 is in good agreement with the experimental results under creep.

The values of the stress exponent and the activation energy at higher stresses in this alloy suggest that the rate-controlling mechanism is the viscous motion of dislocations (solute drag mechanism) [37]. This conclusion is the same as that drawn by He et al. [21] for the identical CoCrFeNiMn alloy (prepared by ingot metallurgy), which they studied via high-temperature (constant strain rate) tensile tests under the conditions they designated as Region I. The conditions of this region corresponded to those of the higher stresses in our investigation. It should be noted that He et al. did not perform tests at a strain rate lower than 6.41 × 10^−7^ s^−1^, i.e., under the conditions where high exponent values were observed in our study. The question of why the dragging behavior manifests itself in the alloy with BN particles and not in other alloys remains open. This may be connected to some subtleties that influence the transition from the climb to the glide of dislocations, e.g., atomic size misfit, and to stacking fault energy [23]. Another factor worthy of consideration is the sub-grain size and its stability. It was shown by Sherby et al. [38] that a stress exponent of 8 was predicted for materials with a stable microstructure. A similar value of stress exponent is observed in the present study of HEA, in both single-phased and dispersion-strengthened states. The blocking of sub-grain migration, either by the presence of particles or due to lattice distortions, may thus directly explain the large values of stress exponents. An identification of the most realistic model describing the observed behavior requires a detailed analysis of the microstructure; this work is currently in progress.

## 5. Conclusions

The results of high-temperature creep tests performed on CoCrFeNiMn alloy containing particles of aluminum nitride or boron nitride showed significant differences in the effect on the creep rate. While aluminum nitride particles have only a small effect on the creep rate, boron nitride particles reduce this rate at high stresses. In contrast, the previously studied oxide particles reduce the creep rate over the entire stress range.

## Figures and Tables

**Table 1:** Compositions of powders used for alloy preparation and of the final compacts after spark plasma sintering. The densities of powders and compacts are shown in the lower part of the table.

Element	HEA-AlN		HEA-BN	
	Powders	SPSed	Powders	SPSed
	(wt %/at %)	(wt %/at %)	(wt %/at %)	(wt %/at %)
Co	21.02/19.73	19.3/17.74	21.02/19.55	19.2/17.3
Cr	18.54/19.73	20.0/20.83	18.54/19.55	19.9/20.32
Fe	19.92/19.73	20.5/19.88	19.92/19.55	20.4/19.4
Mn	19.59/19.73	19.8/19.52	19.5919.55	19.7/19.04
Ni	20.93/19.73	19.0/17.53	20.93/19.55	18.9/17.1
AlN	0.5/1.34			
BN			0.5/2.2	
Al		0.7/1.41		
B				0.7/3.44
N		0.7/2.71		0.8/3.03
Si		0.2/0.39		0.20/0.38
density (g·cm^−3^)	7.96	7.76	7.96	7.71
relative density (%)		97.44		96.74
grain size d_50_ (μm)		0.43		1.53

## Data Availability

The data presented in this study are available on request from the corresponding author. The data are not publicly available due to ongoing research.

## References

[1] Yeh J.W., Chen S.K., Lin S.J., Gan J.Y., Chin T.S., Shun T.T., Tsau C.H., Chang S.Y. (2004). Nanostructured high-entropy alloys with multiple principal elements: Novel alloy design concepts and outcomes. Adv. Eng. Mater..

[2] Tsai M.H., Yeh J.W. (2014). High-Entropy Alloys: A Critical Review. Mater. Res. Lett..

[3] Zhang Y., Zuo T.T., Tang Z., Gao M.C., Dahmen K.A., Liaw P.K., Lu Z.P. (2014). Microstructures and properties of high-entropy alloys. Prog. Mater. Sci..

[4] Pickering E.J., Jones N.G. (2016). High-entropy alloys: A critical assessment of their founding principles and future prospects. Inter. Mater. Rev..

[5] Miracle D.B., Senkov O.N. (2017). A critical review of high entropy alloys and related concepts. Acta Mater..

[6] Lyu Z., Lee C., Wang S.Y., Fan X., Yeh J.W., Liaw P.K. (2019). Effects of Constituent Elements and Fabrication Methods on Mechanical Behavior of High-Entropy Alloys: A Review. Metall. Mater. Trans. A.

[7] Manzoni A.M., Glatzel U. (2019). New multiphase compositionally complex alloys driven by the high entropy alloy approach. Mater. Charact..

[8] George E.P., Raabe D., Ritchie R.O. (2019). High-entropy alloys. Nat. Rev. Mater..

[9] Torralba J.M., Alvaredo P., García-Junceda A. (2019). High-entropy alloys fabricated via powder metallurgy. A critical review. Powder Metall..

[10] Praveen S., Kim H.S. (2018). High-Entropy Alloys: Potential candidates for high-temperature applications—An overview. Adv. Eng. Mater..

[11] Chen J., Zhou X., Wang W., Liu B., Lv Y., Yang W., Xu D., Liu Y. (2018). A review on fundamental of high entropy alloys with promising high-temperature properties. J. Alloys Compd..

[12] Chokshi A.H. (2018). High temperature deformation in fine grained high entropy alloys. Mater. Chem. Phys..

[13] Han L., Xu X., Wang L., Pyczak F., Zhou R., Liu Y. (2019). A eutectic high-entropy alloy with good high-temperature strength-plasticity balance. Mater. Res. Lett..

[14] George E.P., Curtin W.A., Tasan C.C. (2020). High entropy alloys: A focused review of mechanical properties and deformation mechanisms. Acta Mater..

[15] Cantor B., Chang I.T.H., Knight P., Vincent A.J.B. (2004). Microstructural development in equiatomic multicomponent alloys. Mater. Sci. Eng. A.

[16] Gali A., George E.P. (2013). Tensile properties of high- and medium-entropy alloys. Intermetallics.

[17] Stepanov N.D., Shaysultanov D.G., Yurchenko N.Y., Zherebtsov S.V., Ladygin A.N., Salishchev G.A., Tikhonovsky M.A. (2015). High temperature deformation behavior and dynamic recrystallization in CoCrFeNiMn high entropy alloy. Mater. Sci. Eng. A.

[18] Reddy S.R., Bapari S., Bhattacharjee P.P., Chokshi A.H. (2017). Superplastic-like flow in a fine-grained equiatomic CoCrFeMnNi high-entropy alloy. Mater. Res. Lett..

[19] Eleti R.R., Bhattacharjee T., Zhao L., Bhattacharjee P.P., Tsuji N. (2018). Hot deformation behavior of CoCrFeMnNi FCC high entropy alloy. Mater. Chem. Phys..

[20] Zhang M., George E.P., Gibeling J.C. (2021). Elevated-temperature deformation mechanisms in a CrMnFeCoNi high-entropy alloy. Acta Mater..

[21] He J.Y., Zhu C., Zhou D.Q., Liu W.H., Nieh T.G., Lu Z.P. (2014). Steady state flow of the FeCoNiCrMn high entropy alloy at elevated temperatures. Intermetallics.

[22] Cao C., Fu J., Tong T., Hao Y., Gu P., Hao H., Peng L. (2018). Intermediate-temperature creep deformation and microstructural evolution of an equiatomic fcc-structured CoCrFeNiMn high-entropy alloy. Entropy.

[23] Kang Y.B., Shim S.H., Lee K.H., Hong S.I. (2018). Dislocation creep behavior of CoCrFeMnNi high entropy alloy at intermediate temperatures. Mater. Res. Lett..

[24] Dobeš F., Hadraba H., Chlup Z., Dlouhý A., Vilémová M., Matějíček J. (2018). Compressive creep behavior of an oxide-dispersion-strengthened CoCrFeMnNi high-entropy alloy. Mater. Sci. Eng. A.

[25] Zhang M., George E.P., Gibeling J.C. (2021). Tensile creep properties of a CrMnFeCoNi high-entropy alloy. Scripta Mater..

[26] Reppich B., Cahn R.W., Haasen P., Kramer E.J. (1993). Particle strengthening. Materials Science and Technology.

[27] Lagneborg R., Bergman B. (1976). The stress/creep rate behaviour of precipitation-hardened alloys. Met. Sci..

[28] Blum W., Reppich B., Wilshire B., Evans R.W. (1985). Creep of Particle-Strengthened Alloys. Creep Behavior of Crystalline Solids.

[29] Rösler J., Arzt E. (1990). A new model-based creep equation for dispersion strengthened materials. Acta Metal. Mater..

[30] Zhang M., Broyles S.E., Gibeling J.C. (2020). An improved description of creep in dispersion-strengthened metals. Acta Mater..

[31] Amar A., Li J.F., Xiang S., Liu X., Zhou Y.Z., Le G.M., Wang X.Y., Qu F.S., Ma S.Y., Dong W.M. (2019). Additive manufacturing of high-strength CrMnFeCoNi-based high entropy alloys with TiC addition. Intermetallics.

[32] Huang S., Wu H., Zhu H., Xie Z., Cheng J. (2022). Enhanced tensile properties of CrMnFeCoNi0.8 high entropy alloy with in-situ TiC particles. Intermetallics.

[33] Li B., Qian B., Xu Y., Liu Z.Y., Xuan F.Z. (2019). Fine-structured CoCrFeNiMn high-entropy alloy matrix composite with 12 wt% TiN particle reinforcements via selective laser melting assisted additive manufacturing. Mater. Lett..

[34] Hadraba H., Chlup Z., Dlouhy A., Dobes F., Roupcova P., Vilemova M., Matejicek J. (2017). Oxide dispersion strengthened CoCrFeNiMn high-entropy alloy. Mater. Sci. Eng. A.

[35] Tsai K.-Y., Tsai M.-H., Yeh J.-W. (2013). Sluggish diffusion in Co–Cr–Fe–Mn–Ni high-entropy alloys. Acta Mater..

[36] Langdon T.G. (1994). A unified approach to grain boundary sliding in creep and superplasticity. Acta Metall. Mater..

[37] Mohamed F.A., Langdon T.G. (1974). The transition from dislocation climb to viscous glide in creep of solid solution alloys. Acta Metall..

[38] Sherby O.D., Klundt R.H., Miller A.K. (1977). Flow stress, subgrain size, and subgrain stability at elevated temperature. Metall. Trans. A.

